# Validation of the 8^th^ edition of the American Joint Committee on Cancer Pathological Prognostic Staging for young breast cancer patients

**DOI:** 10.18632/aging.103111

**Published:** 2020-04-22

**Authors:** Juan Zhou, Jian Lei, Jun Wang, Chen-Lu Lian, Li Hua, Li-Chao Yang, San-Gang Wu

**Affiliations:** 1 Department of Obstetrics and Gynecology, The First Affiliated Hospital of Xiamen University, Xiamen 361003, People’s Republic of China; 2 Department of Radiation Oncology, The First Affiliated Hospital of Xiamen University, Xiamen 361003, People’s Republic of China; 3 Xiamen Key Laboratory of Chiral Drugs, Medical College, Xiamen University, Xiamen 361005, People’s Republic of China

**Keywords:** breast neoplasms, neoplasm staging, prognosis, AJCC 8 edition ^th^

## Abstract

Purpose: This study aimed to validate the newly proposed American Joint Committee on Cancer (AJCC) pathological prognostic staging system for young breast cancer patients (aged ≤40 years).

Results: We included 12811 women in this study. Overall, 52.8% of patients in the 7^th^ AJCC stages were restaged to the 8^th^ AJCC pathological staging system, including 10.7% upstaged and 42.1% downstaged. The receiver operating characteristics analysis showed that the new staging system had a better role in predicting breast cancer-specific survival (BCSS) compared with 7^th^ edition staging (P<0.001). The results of the multivariate prognostic analysis showed that the hazard ratio of BCSS increased with the 8^th^ AJCC stages, while the 7^th^ anatomic stages had no significant difference in BCSS.

Conclusions: The novel pathological staging system could provide more accurate prognostic stratification for young women with breast cancer because of the high proportion of stage migration.

Patients and Methods: Data for young breast cancer patients diagnosed between 2010 and 2014 were included from the Surveillance, Epidemiology, and End Results program. Chi-squared test, Kaplan–Meier method, receiver operating characteristics curve, and Cox proportional hazard analysis were applied to statistical analysis.

## INTRODUCTION

Breast cancer is mainly an aging disease, with only 5%-7% of new breast cancer cases diagnosed in young women (aged ≤40 years) in the developed world [1, 2]. However, a higher proportion of Asian women were diagnosed at a young age, especially in China, which could reach up to 22% in newly diagnosed breast cancer cases [3–5]. Young breast cancer patients represent a unique subgroup, including more advanced stages and have more aggressive tumor biology including higher tumor grade, hormone receptor negative, human epidermal growth factor receptor-2 (HER2) positive, lymphovascular invasion compared to their older counterparts [6, 7]. Aggressive biologic features have been related to inferior outcomes [1, 8–10]. However, even after adjustment for the factors mentioned above, young age remains a significant adverse prognostic factor for breast cancer-related death [11–14]. As a higher proportion of young women are diagnosed with breast cancer in developing countries, breast cancer will undoubtedly be the leading cause of cancer-related deaths and have a significant burden.

The traditional anatomic tumor (T), node (N), and metastasis (M) staging system was essential for prognostic assessment and treatment decision-making in breast cancer [15]. As the understanding of heterogeneous features in breast cancer has evolved, the lack of biologic information in the traditional American Joint Committee on Cancer (AJCC) anatomic stages has become a limitation in its prognostic assessment [15]. In light of this, the most recent AJCC 8^th^ edition of the pathological prognostic staging system had incorporated the biologic features including histologic grade, estrogen receptor (ER), progesterone receptor (PR), and HER2 status [16, 17]. Several recent studies have validated the prognostic effect of the new staging system in breast cancer [18–20]. However, the prognostic impact of tumor biologic factors incorporated in the current staging system of young patients remains controversial [21–33]. Several prior studies showed similar or inferior outcomes related to ER-positive status in young women [21–27], while others indicated better survival rates associated with ER-positive status [28, 29]. Moreover, the effect of HER2 status on outcomes of breast cancer had an impact on the receipt of anti-HER2 therapy. HER2 positive status was associated with inferior outcomes before the era of anti-HER2 therapy, while it was related to better survival during the era of anti-HER2 therapy [30–33]. Therefore, it is not clear whether the newly proposed staging system holds true for young patients. In light of this, we conducted this validation study to evaluate the newly proposed pathological prognostic stages in young breast cancer patients using a large cohort form Surveillance, Epidemiology, and End Results (SEER) database.

## RESULTS

### Patient Characteristics

**Figure 1:**
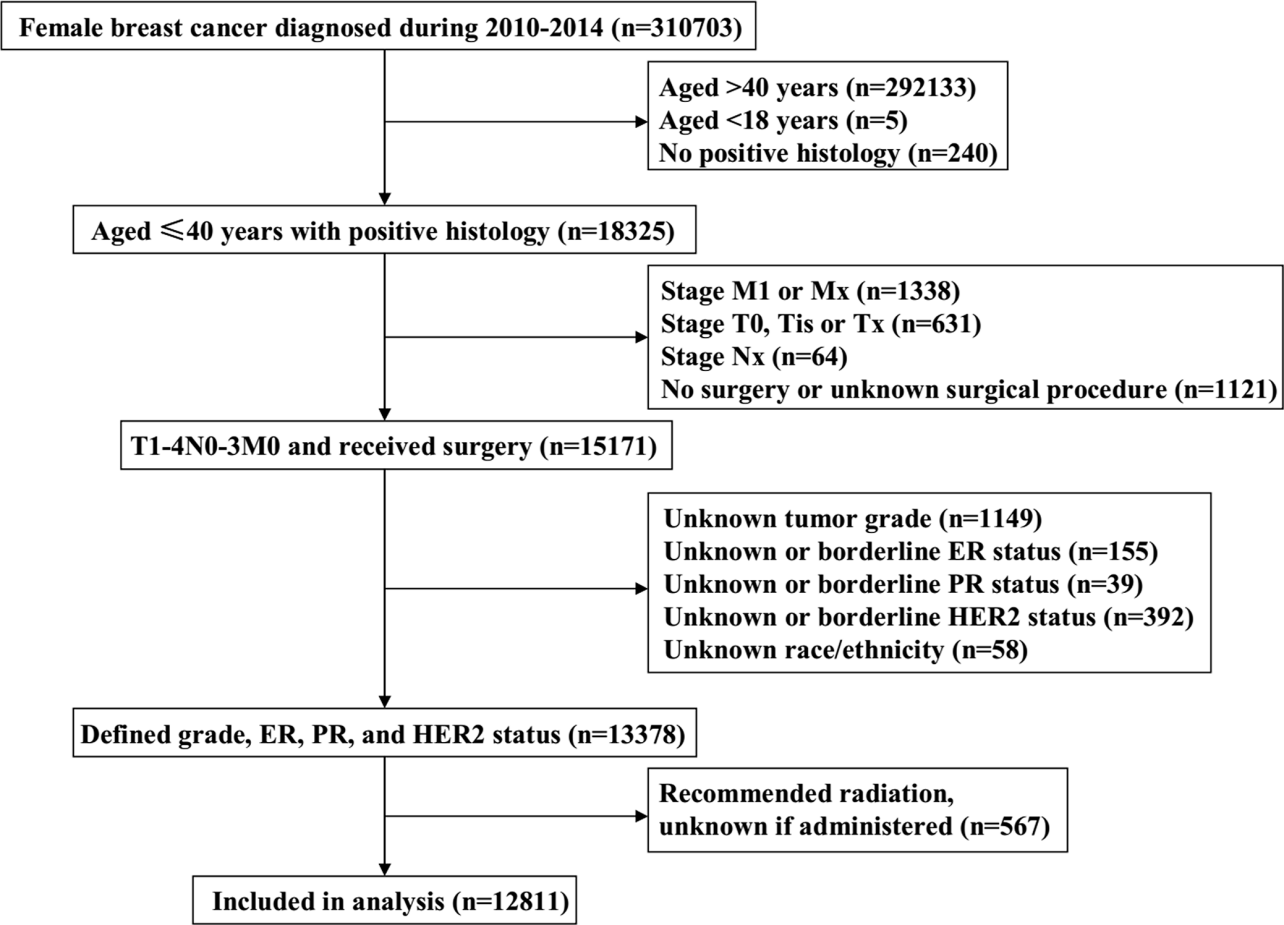
**The patient selection flowchart of the study.**

Overall, 12811 women were included and assigned to both the 7^th^ AJCC anatomic stages and the 8^th^ AJCC pathological prognostic stages. Figure 1 depicts the patient selection flowchart for this study. Patient baseline characteristics are summarized in Table 1. The majority of women were aged 31-40 years (88.1%), with infiltrating ductal carcinoma (IDC) subtype (84.6%), stage T1-2 (86.4%), ER positive (72.7%), and HER2 negative (76.3%) disease. Pathological nodal stages included 53.5%, 33.7%, 8.5% and 4.4% in N0, N1/N1mi, N2, and N3, respectively.

**Table 1:** Patient baseline characteristics.

**Variables**	**n (%)**
Age (years)	
18-30	1528 (11.9)
31-40	11283 (88.1)
Race/ethnicity	
Non-Hispanic White	6891 (53.8)
Non-Hispanic Black	1803 (14.1)
Hispanic (All Races)	2444 (19.1)
Other	1673 (13.1)
Histological subtype	
Infiltrating ductal carcinoma	10836 (84.6)
Invasive lobular carcinoma	353 (2.8)
Other	1622 (12.7)
T stage	
T1	5582 (43.6)
T2	5480 (42.8)
T3	1364 (10.6)
T4	385 (3.0)
N stage	
N0	6848 (53.5)
N1	4317 (33.7)
N2	1086 (8.5)
N3	560 (4.4)
Grade	
Well differentiated	1191 (9.3)
Moderately differentiated	4584 (35.8)
Poorly/undifferentiated	7036 (54.9)
ER status	
Negative	3502 (27.3)
Positive	9309 (72.7)
PR status	
Negative	4577 (35.7)
Positive	8234 (64.3)
HER2 status	
Negative	9773 (76.3)
Positive	3038 (23.7)
Local treatment	
BCS+RT	3033 (23.7)
BCS alone	895 (7.0)
MAST	5328 (41.6)
MAST+RT	3555 (27.7)
Chemotherapy	
No	2863 (22.3)
Yes	9948 (77.7)

BCS, breast-conservation surgery; ER, estrogen receptor; HER2, human epidermal growth factor receptor 2; PR, progesterone receptor; MAST, mastectomy; N, nodal; RT, radiotherapy; T, tumor.

Regarding local and systemic treatments, 69.3% of patients underwent mastectomy and 30.7% of them were treated with lumpectomy. Of the lumpectomy patients, 77.2% received adjuvant radiotherapy, whereas 40.0% of patients were treated with adjuvant radiotherapy following mastectomy. Of the entire cohort, 77.7% of patients received chemotherapy.

### Restaging

The stage-by-stage differences between the AJCC 7^th^ and 8^th^ edition-based stages are summarized in Table 2. Significant differences were found in the stage breakdown between the two edition staging systems (P<0.001). In the entire cohort, 52.8% of patients in the 7^th^ AJCC staging system were restaged to the 8^th^ AJCC pathological prognostic staging system, including 10.7% upstaged and 42.1% downstaged (Table 2). Overall, 92.0% of patients in the 7^th^ edition stage IB disease were downstaged to IA disease according to the 8^th^ edition criteria. In addition, 57.3%, 60.4%, 64.1%, and 74.8% of the 7^th^ edition stage IIA, IIB, IIIA, and IIIC patients were also significantly downstaged, respectively (Table 2).

**Table 2:** The stage-by-stage differences between the 7^th^ and 8^th^ edition staging systems.

**8^th^ AJCC pathological prognostic staging system**									
**7^th^ AJCC anatomic staging system**	IA (%)	IB (%)	IIA (%)	IIB (%)	IIIA (%)	IIIB (%)	IIIC (%)	Total
	IA	3359 (85.8)	557 (14.2)	0	0	0	0	0	3916
	IB	297 (92.0)	26 (8.0)	0	0	0	0	0	323
	IIA	1411 (39.4)	642 (17.9)	1532 (42.7)	0	0	0	0	3585
	IIB	99 (4.0)	970 (38.9)	436 (17.5)	531 (21.3)	460 (18.4)	0	0	2496
	IIIA	0	536 (33.2)	152 (9.4)	347 (21.5)	326 (20.2)	18 (1.1)	236 (14.7)	1615
	IIIB	0	0	0	0	63 (19.9)	148 (46.8)	105 (33.2)	316
	IIIC	0	0	0	0	145 (25.9)	274 (48.9)	141 (25.2)	560
	Total	5166 (40.3)	2731 (21.3)	2120 (16.5)	878 (6.9)	994 (7.8)	440 (3.4)	482 (3.8)	12811

Orange boxes, light green boxes, and blue boxes represent those who were upstaged, downstaged, and unchanged, respectively.

For patients with IDC, 13.0% were upstaged and 40.7% were downstaged, whereas 1.1% of invasive lobular carcinoma patients were upstaged and 62.6% were downstaged (P <0.001). For patients with stage N1, N2, and N3 diseases, 64.6%, 64.0%, and 74.8% of them were downstaged (P <0.001). For patients with poorly/undifferentiated disease, 34.1% of them were downstaged, and 18.3% were upstaged. Patients with ER (38.8% vs. 2.7%, P<0.001) and PR (29.9% vs. 4.3%, P<0.001) negative disease had a more proportion of upstaging compared to those with ER and PR positive diseases. Moreover, 14.1% of HER2 negative disease were upstaged, whereas no patients were upstaged in HER2 positive patients (P<0.001) (Table 3).

**Table 3:** Demographic and tumor characteristics by stage change from the 7^th^ to the 8^th^ edition of the AJCC breast cancer staging manual.

**Variables**	**Downstage (%)**	**No change (%)**	**Up stage (%)**	**P**
Age (years)				
18-30	662 (40.7)	708 (46.3)	198 (13.0)	0.012
31-40	4750 (42.1)	5355 (47.5)	1178 (10.4)	
Race/ethnicity				
Non-Hispanic White	2914 (42.3)	3308 (48.0)	669 (9.7)	<0.001
Non-Hispanic Black	666 (36.9)	863 (47.9)	274 (15.2)	
Hispanic (All Races)	1050 (43.0)	1102 (45.1)	292 (11.9)	
Other	742 (44.4)	790 (47.2)	141 (8.4)	
Histological subtype				
Infiltrating ductal carcinoma	4419 (40.8)	5176 (47.8)	1241 (11.5)	<0.001
Invasive lobular carcinoma	221 (62.6)	128 (36.3)	4 (1.1)	
Other	732 (45.1)	759 (46.8)	131 (8.1)	
T stage				
T1	1255 (22.5)	3748 (67.1)	579 (10.4)	<0.001
T2	3215 (58.7)	1817 (33.2)	448 (8.2)	
T3	794 (58.2)	326 (23.9)	244 (17.9)	
T4	108 (28.1)	172 (44.7)	105 (27.3)	
N stage				
N0	1470 (21.5)	4696 (68.6)	682 (10.0)	<0.001
N1	2788 (64.6)	999 (23.1)	530 (12.3)	
N2	695 (64.0)	227 (20.9)	164 (15.1)	
N3	419 (74.8)	141 (25.2)	0 (0)	
Grade				
Well differentiated	459 (38.5)	732 (61.5)	0 (0)	<0.001
Moderately differentiated	2515 (54.9)	1979 (43.2)	90 (2.0)	
Poorly/undifferentiated	2398 (34.1)	3352 (47.6)	1286 (18.3)	
ER status				
Negative	94 (2.7)	2048 (58.5)	1360 (38.8)	<0.001
Positive	5278 (56.7)	4015 (43.1)	16 (0.2)	
PR status				
Negative	197 (4.3)	3010 (65.8)	1370 (29.9)	<0.001
Positive	5175 (62.8)	3053 (37.1)	6 (0.1)	
HER2 status				
Negative	3985(40.8)	4412 (45.1)	1376 (14.1)	<0.001
Positive	1387(45.7)	1651 (54.3)	0 (0)	

ER, estrogen receptor; HER2, human epidermal growth factor receptor 2; PR, progesterone receptor; N, nodal; T, tumor.

### BCSS between the 7^th^ and 8^th^ edition of staging systems

**Figure 2:**
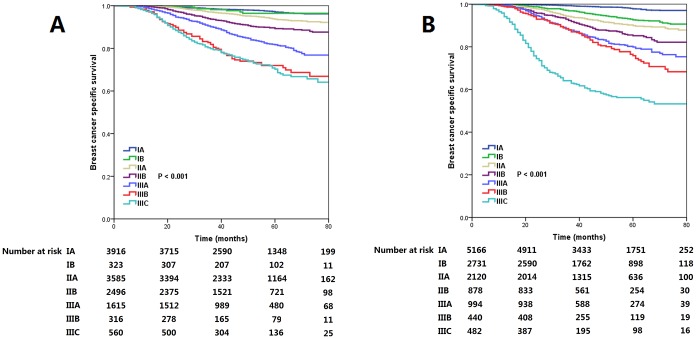
The breast cancer specific survival curves by the 7^th^ (**A**) and 8^th^ (**B**) edition of the AJCC staging systems.

**Figure 3:**
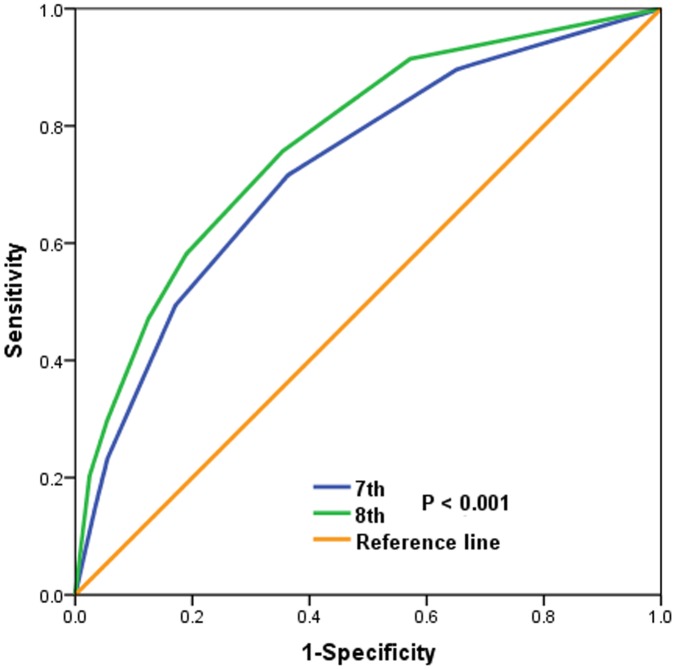
**ROC analyses for prediction the breast cancer specific survival by the 7^th^ and 8^th^ edition of the AJCC staging systems.**

With a median follow-up of 47 months, 1069 patients died, and 934 (87.4%) of them died with breast cancer-related disease. A significant difference was found in breast cancer-specific survival (BCSS) between the 7^th^ and 8^th^ edition of staging systems. The BCSS curves indicated more overlap lines in the 7^th^ edition stages compared to the 8^th^ edition stages. In the 7^th^ AJCC staging system, curves of stage IA and IB diseases were overlapped, and curves of stage IIIB and IIIC diseases were also overlapped. Significantly differences regarding the BCSS curves were found among the 8^th^ edition stages. The 5-year BCSS rates for the 7^th^ edition stages were 97.0% for stage IA, 96.4% for stage IB, 93.6% for stage IIA, 89.3% for stage IIB, 81.7% for stage IIIA, 71.9% for stage IIIB, and 70.3% for stage IIIC (P<0.001) (Figure 2A). The 5-year BCSS rates for the 8^th^ edition stages were 97.8%, 92.6%, 90.0%, 85.2%, 79.9%, 75.9%, and 56.2% in stage IA, IB, IIA, IIB, IIIA, IIIB, and IIIC diseases, respectively (P<0.001) (Figure 2B). The receiver operating characteristics (ROC) analysis showed that the new staging system had a better role in predicting the BCSS compared to 7^th^ edition stages (area under the curve [AUC]: 0.773 vs. 0.728, P<0.001) (Figure 3).

### Multivariate prognostic analysis

A Cox proportional hazard model was performed to analyze the prognostic effect among all available potential prognostic factors associated with BCSS. The results showed that race/ethnicity, T stage, N stage, tumor grade, ER, PR, and HER2 status were the independent prognostic factors related to BCSS (Table 4).

**Table 4:** Multivariate prognostic analysis including available potential prognostic factors.

**Variables**	**HR**	**95%CI**	**P**
Age (years)			
18-30	1		
31-40	0.924	0.770-1.109	0.396
Race/ethnicity			
Non-Hispanic White	1		
Non-Hispanic Black	1.407	1.192-1.661	<0.001
Hispanic (All Races)	1.262	1.070-1.489	0.006
Other	0.795	0.625-1.013	0.063
Histological subtype			
Infiltrating ductal carcinoma	1		
Lobular carcinoma	1.430	0.961-2.126	0.078
Other	0.812	0.656-1.005	0.056
T stage			
T1	1		
T2	1.560	1.308-1.861	<0.001
T3	2.492	2.022-3.071	<0.001
T4	4.338	3.361-5.600	<0.001
N stage			
N0	1		
N1	2.124	1.799-2.507	<0.001
N2	3.959	3.239-4.840	<0.001
N3	5.680	4.561-7.075	<0.001
Grade			
Well differentiated	1		
Moderately differentiated	2.038	1.237-3.359	0.005
Poorly/undifferentiated	3.316	2.020-5.442	<0.001
ER status			
Negative	1		
Positive	0.600	0.493-0.731	<0.001
PR status			
Negative	1		
Positive	0.636	0.520-0.779	<0.001
HER2 status			
Negative	1		
Positive	0.464	0.389-0.553	<0.001

CI, confidence interval; ER, estrogen receptor; HER2, human epidermal growth factor receptor 2; HR, hazard ratio; PR, progesterone receptor; N, nodal; T, tumor.

Next, we also performed a Cox proportional hazard analysis to compare the prognostic effect of the two stage groups on BCSS. The results showed that the 8^th^ AJCC pathological prognostic staging system had superior overall discriminatory power to predict the BCSS. The hazard ratio (HR) of BCSS increased with the staging. When using stage IA as the reference, all categories in the 8^th^ AJCC pathological prognostic stages showed worse BCSS with gradually increased HRs. In contrast, the 7^th^ anatomic stages had no significant difference in BCSS using the multivariate prognostic analysis (Table 5).

**Table 5:** Multivariate prognostic analysis including the 7^th^ and 8^th^ edition staging systems.

**Variables**	**HR**	**95%CI**	**p**
Age (years)			
18-30	1		
31-40	0.925	0.771-1.110	0.402
Race/ethnicity			
Non-Hispanic White	1		
Non-Hispanic Black	1.432	1.213-1.690	<0.001
Hispanic (All Races)	1.300	1.102-1.534	0.002
Other	0.808	0.635-1.029	0.084
Histological subtype			
Infiltrating ductal carcinoma	1		
Invasive lobular carcinoma	1.454	0.987-2.141	0.058
Other	0.841	0.680-1.039	0.109
7^th^ AJCC anatomic stages			
IA	1		
IB	1.165	0.613-2.611	0.526
IIA	0.792	0.569-1.101	0.165
IIB	0.816	0.580-1.147	0.241
IIIA	0.900	0.627-1.292	0.568
IIIB	0.986	0.628-1.546	0.950
IIIC	1.154	0.755-1.764	0.508
8^th^ AJCC pathological prognostic stages			
IA	1		
IB	3.474	2.645-4.562	<0.001
IIA	5.032	3.847-6.582	<0.001
IIB	7.821	5.841-10.473	<0.001
IIIA	10.983	8.395-14.370	<0.001
IIIB	13.468	9.938-18.253	<0.001
IIIC	32.847	25.226-42.771	<0.001

CI, confidence interval; ER, estrogen receptor; HER2, human epidermal growth factor receptor 2; HR, hazard ratio; PR, progesterone receptor; N, nodal; T, tumor.

## DISCUSSION

T stage and N stage remain the essential indicators influencing the survival outcome of breast cancer [15]. Before the 8^th^ breast cancer prognostic stages were proposed, the traditional anatomic stages had been widely used to predict the prognosis and guide adjuvant treatment decisions. However, the traditional anatomic TNM stages might not be sufficient to reflect the survival of all patients and draw up the subsequent decision-making process. To provide more accurate prognostic information for breast cancer patients, the novel pathological prognostic stages have incorporated the ER, PR, HER2, and grade into the anatomic TNM stages [16, 17]. In our study, we validated the prognostic performance of 8^th^ AJCC pathological prognostic stages in young breast cancer patients. In BCSS analyses, the 8^th^ edition of AJCC prognostic stages had a better distinguish of survival compared to the traditional anatomic staging, suggesting that the newly proposed pathological prognostic stages also have prognostic significance in young patients. Our study showed that the 8^th^ edition of AJCC pathological prognostic staging was also true when adjusted by young breast cancer patients.

We sought to investigate how many patients would be restaged in the pathological prognostic stages compared to the traditional anatomic stages. A recent study from National Cancer Data Base (n=493854) including all ages of patients, patients were diagnosed with early T stage (T1, 70.6%; T2, 25.6%; T3, 3.1%; T4, 0.7%) and N stage (N0, 75.3%; N1, 18.3%; N2, 4.4%; N3, 2.0%) compared to our study, with stage changed in 36.6% of patients (6.8% upstaged and 29.7% downstaged) [18]. A prior SEER study (n=168076) indicated that 53.2% of patients were restaged, 22.1% of patients downstaged and 31.2% of patients were upstaged [19]. In addition, a large cohort included patients from Korea (n=24014), 45.5% of patients were restaged, including 26.1% were upstaged and 19.4% were downstaged [20]. In our study, only 43.6% and 53.5% of patients were T1 stage and node-negative disease, respectively. Of the entire cohort, 52.8% of patients were restaged, including 42.1% were downstaged and 10.7% were upstaged when pathological prognostic stages were compared to anatomic stages. The differences in the distributions of the anatomic stage between young and their older counterparts may possibly explain the higher percentage of patients downstaged in young patients.

It does appear that conflicting results regarding the role of tumor biology, including tumor grade, ER, PR, and HER2 status in young breast cancer. Several prior studies showed similar or inferior outcomes related to ER positive status in young patients [21–27], while others demonstrated superior outcomes associated with ER positive status [28, 29]. It was hypothesized that young patients have lower compliance with hormonal therapy [34]. However, patients who were untreated with systemic therapy also showed inferior survival outcomes in young luminal-B patients [14]. In addition, the prognostic effect of HER2 status on survival outcomes had an impact on the receipt of the anti-HER2 therapy [29–33]. A study from Japan showed that the triple-negative breast cancer had the worst outcomes in patients aged ≤40 years, while comparable outcomes were found among the luminal A, luminal B, and HER2 overexpression subtypes [35]. Similar results were also found from the European Organisation for Research and Treatment of Cancer clinical trials [36]. However, the data on tamoxifen use was largely missing in this study, and anti-HER2 therapy was also not recorded. In the present study, patients with hormone receptor positive disease had better BCSS compared to those with hormone receptor negative disease, and we believed this was due to the advances of hormone therapy. In addition, our study also indicated that HER2-positive patients had significantly better BCSS compared to those with HER2-negative disease, which was also due to the progress of anti-HER2 treatment.

Since most patients determined in the 8^th^ edition of the AJCC stages received multimodal therapy, including chemotherapy, hormone therapy, and anti-HER2 targeted therapy. In the current practice, patients with ER positive, PR positive, and HER2 positive were more likely to be assigned to lower stages when other staging factors were the same, while patients with ER negative, PR negative, and HER2 negative status were more likely to be assigned to higher stages. Therefore, the prognosis reflected by the new staging system was the prognosis after comprehensive standardized treatment based on the patient's clinical and biologic characteristics [16, 17]. Patients with lower stage did not mean that the patients needed less treatment, but reflected that the patients had better biologic characteristics or more effective treatment. Although patients included in our study did not have the record on hormone therapy and anti-HER2 therapy, our patients were in the era of contemporary treatment. In addition, this study confirmed that pathological prognostic stages provided more accurate information on survival compared to the anatomic stage. However, it should be noted that the AJCC 8^th^ edition stages could accurately assess the prognosis of patients based on the routine application of anti-HER2 therapy. Therefore, the application of the newly proposed AJCC staging system should be minded in countries or regions where anti-HER2 therapy is still expensive and cannot be widely used.

A previous SEER study showed that at 0-5 years after diagnosis, ER negative patients had a higher risk of breast cancer-specific mortality than ER positive patients. However, at 5-10 years after diagnosis, ER positive disease had increased risk of breast cancer-specific mortality compared to ER negative patients [28]. Similar results from the Prospective Study of Outcomes in Sporadic and Hereditary Breast Cancer (POSH) study also showed better 5-year distant recurrence-free survival for ER positive disease compared to ER negative disease (78.5% vs. 72.5%). However, comparable 8-year distant recurrence-free survival was found between ER positive and ER negative disease [37]. The long-term study from POSH also confirmed patients with ER positive tumors had comparable overall survival compared to those with ER negative tumors in both HER2 positive and HER2 negative subgroups [38]. It should be noted that the median follow-up was only 47 in our study. In addition, the median follow-up was only 37.6 months in patients that included in the determination of the 8^th^ AJCC pathological prognostic staging system [16, 17]. Extended use of hormone therapy has currently recommended in young patients [39, 40], but the use of hormone therapy in our study and the patient's determination of the 8^th^ AJCC pathological prognostic staging system have not yet reached 10-years. Therefore, long-term follow-up results are still needed to verify the effect of the new staging system in young breast cancer patients.

The significant strength of our study is that we used a large population-based cohort to evaluate the prognostic performance of the newly proposed pathological prognostic staging system in younger patients. In addition, the patients included in this study were in the modern treatment mode, which makes our study valuable and unique. However, several limitations should be recognized in our study. First, potential intrinsic bias should not be neglected by the nature of the retrospective studies. Second, the details of hormone therapy, anti-HER2 treatment, and chemotherapy were not recorded in the SEER database, which might impact the prognostic assessment. Third, the length of follow-up was inadequate in our study. Finally, long-term results regarding the outcomes for patients with various pathological prognostic stages are needed to further validate the prognostic performance of newly proposed pathological prognostic staging system in younger patients.

In conclusion, our study suggests that the novel pathological staging system could provide more accurate prognostic stratification for young women with breast cancer because of the high proportion of stage migration. More studies with long-term follow-up are needed to confirm the validity of this staging system and guide treatment-decision making in younger breast cancer patients.

## MATERIALS AND METHODS

### Patients

Young women diagnosed with breast cancer from 2010 to 2014 were identified using the SEER database. SEER database is a population-based cancer registry, which including information on cancer incidence, demographic and tumor features, the first course of treatment, and outcomes for approximately 28% of the United States population [41]. We identified patients who met the following criteria: T1-4N0-3M0 invasive breast cancer; aged ≤40 years; treatment with breast-conservation surgery or mastectomy; available variables including tumor grade, ER status, PR status, HER2 status, and race/ethnicity. Patients with aged <18 years, non-positive pathological diagnoses, and unavailable of local treatment procedures were excluded. The present study was exempted from approval by the Institutional Review Board due to the de-identified information was included in the SEER program.

### Variables

We identified the following patients’ demographic and clinicopathological information: age, race/ethnicity, T stage, N stage, tumor grade, histology, ER, PR, and HER2 status. Moreover, the receipt of chemotherapy and local treatment procedures, including surgery and radiotherapy, were also identified in this study. The pathological prognostic stages were assigned according to the newly proposed AJCC breast cancer pathological prognostic staging manual [16, 17].

### Statistical analysis

Comparisons of the proportions of upstage or downstage classifications between the 7^th^ edition of the AJCC anatomic stages and the 8^th^ edition of the AJCC pathological prognostic stages were performed using the chi-squared test or *fisher*'s exact *test*. BCSS was estimated from the time of diagnosis of breast cancer to the time of death from breast cancer or the follow-up cutoff. Survival curves were plotted using the Kaplan–Meier method and the significant difference among different stages was compared using the log-rank test. The AUC was estimated to compare the model fit using the ROC curve. Cox proportional hazards model was used to determine the independent prognostic factors associated with BCSS. All statistical analyses were conducted by IBM SPSS 22.0 software package (IBM Corp., Armonk, NY). Two-sided P values <0.05 were considered statistically significant.
